# The association between collagenopathies and the risk of spontaneous preterm birth: A systematic review with selective meta‐analysis

**DOI:** 10.1111/aogs.70361

**Published:** 2026-10-09

**Authors:** Kensie Treacy, Brenda F. Narice, Denya Williams‐Goss, Dilly O. C. Anumba

**Affiliations:** ^1^ Division of Clinical Medicine, School of Medicine and Population Health University of Sheffield Sheffield UK; ^2^ Sheffield Teaching Hospitals NHS Foundation Trust Sheffield UK

**Keywords:** collagenopathy, Ehlers–Danlos syndrome, Marfan syndrome, pregnancy, preterm birth

## Abstract

**Introduction:**

Spontaneous preterm birth (sPTB) remains a leading cause of neonatal morbidity and mortality. Inherited collagenopathies, including Ehlers–Danlos syndrome (EDS) and Marfan syndrome (MFS), may predispose to sPTB through impaired connective tissue integrity; however, the available evidence has not been systematically synthesized according to collagenopathy subtype. We aimed to evaluate the association between inherited collagenopathies and sPTB through a systematic review, undertaking quantitative synthesis only where clinically appropriate and methodologically comparable.

**Material and Methods:**

This systematic review followed PRISMA 2020 guidelines and was prospectively registered in PROSPERO (CRD420251021631). MEDLINE (PubMed), Embase (Ovid), Scopus, Web of Science, and the Cochrane Library were searched from inception to 30 April 2025. Observational studies comparing pregnancy outcomes in women with inherited collagenopathies and women without collagenopathies were eligible. The primary outcome was sPTB before 37 weeks' gestation. Findings were synthesized narratively according to collagenopathy subtype. Meta‐analysis using a Mantel–Haenszel fixed‐effect model was undertaken only for clinically comparable MFS studies.

**Results:**

Fourteen observational studies met the inclusion criteria. Four studies evaluated MFS, seven evaluated EDS, one assessed vascular EDS exclusively and two included mixed collagenopathy cohorts. Meta‐analysis demonstrated that women with MFS had significantly higher odds of sPTB than controls (OR 2.71, 95% CI 2.23–3.30; *p* < 0.001; *I* ^2^ = 41%). Studies of EDS demonstrated substantial clinical and methodological heterogeneity, precluding meaningful primary quantitative synthesis. Nevertheless, narrative synthesis consistently identified increased frequencies of sPTB and obstetric complications.

**Conclusions:**

Inherited collagenopathies appear to be associated with an increased risk of sPTB, although the certainty of evidence differs across phenotypes. The strongest comparative evidence currently exists for MFS, whereas evidence for EDS syndrome remains limited by substantial clinical heterogeneity. These findings support phenotype‐specific obstetric assessment and highlight the need for prospective studies with standardized subtype classification and outcome reporting.

## Graphical abstract

This review quantifies the association between collagenopathies and spontaneous preterm birth, assessing their role in antenatal risk assessment. Marfan syndrome is significantly associated with an increased risk of spontaneous preterm birth (OR 2.71, 95% CI 2.23–3.30; *p* < 0.001; *I* ^2^ = 41%) while evidence for Ehlers–Danlos syndrome is heterogeneous, varying by subtype.

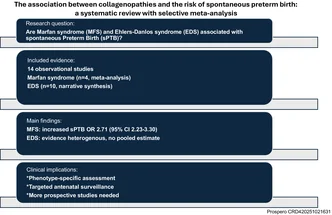

**Abbreviations**
EDS: Ehlers–Danlos syndrome
MFS: Marfan syndrome
PTB: Preterm Birth
sPTB: Spontaneous Preterm Birth

**Key Message**

Evidence suggests an increased risk of spontaneous preterm birth among women with Marfan syndrome; however, evidence for Ehlers–Danlos syndrome and other collagenopathies remains limited and heterogeneous. Further phenotype‐specific research is needed to inform antenatal risk assessment and care.

## 1 INTRODUCTION

Spontaneous preterm birth (sPTB) affects about 8% of births in the UK and remains the leading cause of neonatal mortality, with significant health and socioeconomic consequences.^1^ The cervix plays a central role in maintaining pregnancy by providing structural support through its extracellular matrix, which is rich in collagen. Collagen provides tensile strength and stability, and its remodeling during pregnancy allows for cervical softening and dilation at term.^2^, ^3^ During pregnancy, collagen concentration is decreased, whereas collagen extractability and water content are increased.^4^ So, a low pre‐pregnancy collagen concentration could be problematic. Women who had a short cervix in the second trimester or cervical insufficiency were found to have lower collagen concentrations in a non‐pregnant state.^5^ Similarly, recent research suggests that the cervices of preterm patients exhibit lower collagen density (<5%) compared to those of term patients.^6^ Furthermore, impaired collagen synthesis or structure may compromise cervical integrity, predisposing to cervical insufficiency, premature prelabour rupture of membranes (PPROM) and sPTB.

Inherited connective tissue disorders such as Ehlers–Danlos syndrome (EDS) and Marfan syndrome (MFS) are characterized by abnormalities in collagen and fibrillin synthesis, affecting individuals of all racial and ethnic backgrounds.^7^ Because collagen is present throughout the body, these disorders can impact multiple organ systems, often leading to overlapping clinical features.^8^ The key distinction between EDS and MFS is that EDS is a group of conditions with hypermobility and skin hyperextensibility being key features, while MFS primarily affects the cardiovascular, skeletal, and ocular systems.^9^ The overall prevalence of these collagenopathies is estimated to be 1 in 5000.^10^, ^11^

Inherited connective tissue disorders comprise genetically and clinically distinct conditions characterized by abnormalities in extracellular matrix proteins, including collagen and fibrillin, which may compromise tissue integrity during pregnancy. While rare, they may have significant implications for pregnancy outcomes. Several observational studies have reported increased risks of cervical insufficiency, PPROM and sPTB among women with inherited connective tissue disorders, although findings have been inconsistent, particularly across EDS subtypes.^12^ Despite growing awareness of their potential obstetric impact, collagenopathies are not currently included in UK antenatal risk models for sPTB. A clearer understanding of their association with sPTB could inform targeted surveillance and preventive strategies for affected women.^12^

This systematic review aimed to synthesize the available evidence regarding the association between inherited collagenopathies, particularly EDS and MFS, and sPTB. Where studies were considered sufficiently clinically and methodologically comparable, quantitative synthesis was undertaken. We additionally sought to explore whether the available evidence supports phenotype‐specific obstetric risk assessment in women with inherited connective tissue disorders.

## 2 MATERIAL AND METHODS

### 2.1 Protocol and reporting

This systematic review was conducted in accordance with the Preferred Reporting Items for Systematic Reviews and Meta‐Analyses (PRISMA) 2020 statement and prospectively registered with the International Prospective Register of Systematic Reviews (PROSPERO; CRD420251021631). The review was undertaken according to the pre‐specified protocol. As planned in the registered protocol, quantitative synthesis was performed only when studies were considered sufficiently homogeneous in terms of clinical and methodological characteristics; otherwise, findings were synthesized narratively.

### 2.2 Search strategy

A comprehensive literature search was undertaken in MEDLINE (PubMed), Embase (Ovid), Scopus, Web of Science and the Cochrane Library from database inception to 30 April 2025. The search strategy combined controlled vocabulary (such as Medical Subject Headings [MeSH], where applicable) and free‐text terms relating to inherited collagen disorders (“Ehlers‐Danlos syndrome”, “Marfan syndrome”, “collagenopath*”, “connective tissue disorder”) and sPTB (“preterm birth”, “premature birth”, “premature labour”, “spontaneous preterm birth”). Database‐specific search strategies are presented in Table S2. The search syntax is reported using the conventions of each database and has been formatted for clarity and readability while preserving the original search concepts.

To maximize study identification, reference lists of all included studies were hand searched, forward citation searching was performed and gray literature, including conference proceedings and abstracts, was searched. Searches were restricted to English‐language studies involving human participants. Following completion of the literature search, but before study selection and data extraction, studies published before 2000 were excluded because advances in the diagnosis and obstetric management of inherited connective tissue disorders, particularly MFS, limit the applicability of earlier studies to contemporary clinical practice. This decision was made in consultation with our maternal cardiology collaborators.

### 2.3 Eligibility criteria

Eligibility criteria were defined according to the Population, Exposure, Comparator, Outcomes and Study design (PECOS) framework.

Population: pregnant women with a diagnosis of an inherited collagenopathy.
Exposure: EDS (all reported subtypes) and MFS as specified in the registered protocol. Although the protocol included other inherited connective tissue disorders, including osteogenesis imperfecta, no eligible comparative pregnancy studies meeting the inclusion criteria were identified for these conditions.
Comparator: pregnant women without a diagnosed collagenopathy or the general obstetric population.
Primary outcome: sPTB before 37 completed weeks' gestation.
Secondary outcomes: sPTB before 34 weeks' gestation, PPROM, postpartum hemorrhage (PPH), cervical insufficiency, fetal growth restriction (FGR), or small‐for‐gestational‐age (SGA) infants, mode of delivery and other reported maternal or neonatal adverse outcomes.

Eligible study designs included cohort, case–control, and cross‐sectional studies. Where outcome data or methodological details were unclear or incompletely reported, attempts were made to contact the corresponding authors to obtain additional information. Full‐text articles were obtained through institutional library access and, where necessary, from alternative academic sources. Studies were not excluded solely because of incomplete reporting; instead, all eligible studies were included in the systematic review where sufficient data were available to inform the narrative synthesis. Quantitative synthesis was undertaken only when extractable data for the prespecified outcome of interest were available. Studies reporting only medically indicated (iatrogenic) PTB without separate reporting of sPTB were excluded only when sPTB data could not be extracted or obtained separately.

### 2.4 Study selection

All records were imported into Covidence, where duplicate records were removed before screening. Two reviewers (KT and DW‐G) independently screened titles and abstracts, followed by independent full‐text assessment of potentially eligible studies. Disagreements were resolved through discussion and, where necessary, consultation with a third reviewer (BFN). Reasons for exclusion at the full‐text stage were recorded prospectively and are presented in the PRISMA flow diagram and Table S4.

### 2.5 Data extraction

Data extraction was performed independently by two reviewers using a standardized data extraction form developed a priori. Extracted variables included study design, country, study period, recruitment setting, participant characteristics, collagenopathy subtype, diagnostic criteria, comparator population, outcome definitions, gestational age thresholds, maternal and neonatal outcomes, reported effect estimates, and variables included in adjusted analyses where applicable. Any discrepancies were resolved through discussion with a third reviewer.

### 2.6 Quality assessment

Methodological quality was independently assessed by two reviewers using the Newcastle–Ottawa Scale for observational studies. Risk‐of‐bias assessments informed interpretation of study findings and decisions regarding quantitative synthesis but were not used as the sole criterion for excluding studies from the review.

### 2.7 Data synthesis and statistical analysis

Clinical and methodological heterogeneity was assessed before undertaking quantitative synthesis, including collagenopathy subtype, study design, comparator population and outcome definitions. Quantitative synthesis was undertaken only when studies were considered sufficiently comparable clinically and methodologically to yield meaningful pooled estimates.

Because inherited collagenopathies represent biologically distinct disorders with differing obstetric phenotypes, the primary quantitative synthesis was restricted to studies of MFS representing the only subgroup judged to have sufficient clinical homogeneity and comparable outcome reporting. Pooled odds ratios (ORs) with 95% confidence intervals (CIs) were calculated using the Mantel–Haenszel fixed‐effect model in RevMan Web (version 5.8). A fixed‐effect model was selected because the objective was to estimate a common effect within this predefined, clinically comparable population after restricting quantitative synthesis to studies with similar clinical characteristics.

Studies evaluating EDS demonstrated substantial heterogeneity in subtype classification, diagnostic criteria, outcome definitions and clinical management. Consequently, these studies were synthesized narratively. Exploratory random‐effects analyses of EDS studies are presented in the Figures S1 and S2 for completeness. These analyses should be interpreted cautiously and were not used to inform the primary conclusions because of substantial clinical and methodological heterogeneity.

Statistical heterogeneity was assessed using the *I* ^2^ statistic and interpreted alongside clinical and methodological heterogeneity. Decisions regarding quantitative synthesis were therefore based on both statistical measures and clinical judgment. Owing to the small number of studies included in each meta‐analysis, publication bias was not formally assessed.

## 3 RESULTS

### 3.1 Study selection

A total of 905 records were identified across all databases. After removing 319 duplicates, 586 articles were screened by title and abstract. Of these, 400 were excluded, and 171 were excluded after full‐text review. After further consideration, one study was excluded^13^ from the analysis due to duplicate studies using the same HCUP databases.^13^, ^14^ The screening process is summarized in Figure 1.

**FIGURE 1:**
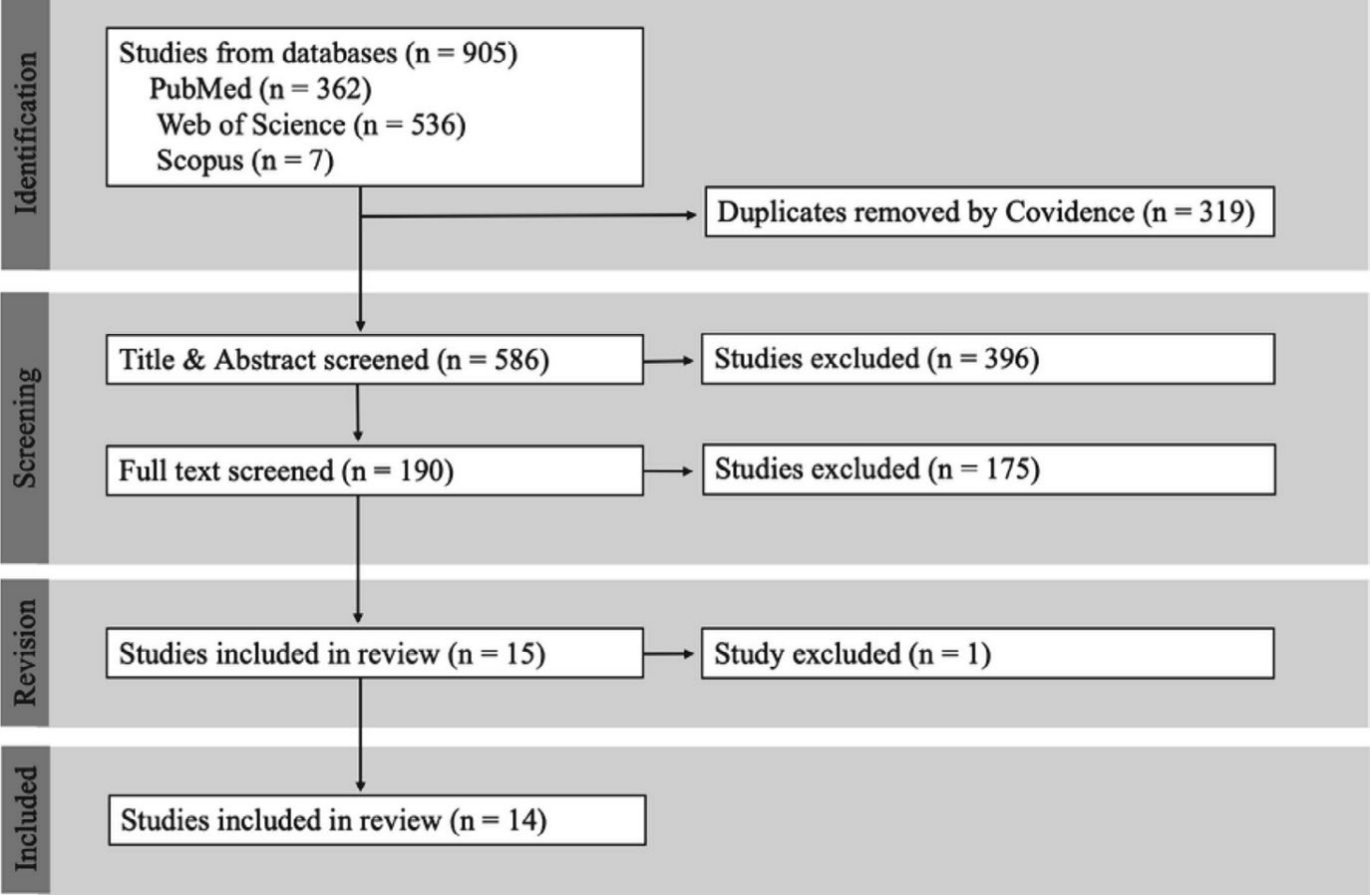
Flow chart to demonstrate the process taken to conduct the systematic review, including the identification and screening phases and accountability for the excluded studies.

### 3.2 Study characteristics

Fourteen observational studies published between 2002 and 2024 met the inclusion criteria (Table 1). Together, these studies included 3982 pregnancies and 9208 births among women with inherited collagenopathies. Four studies evaluated MFS, seven evaluated EDS, one evaluated vascular EDS exclusively, and two included mixed collagenopathy cohorts. Key study characteristics are summarized in Table 1, with detailed study characteristics and findings presented in Tables S5 and S6.

**TABLE 1:** Study characteristics of the included studies.

Study	Country	Study design	Setting	Affected (*n*)	Comparator (*n*)	Subtype	Outcome	NOS
Underhill et al.	USA	Cross‐sectional	Patient registry	1068	191	Mixed EDS	sPTB/PTB	4
Kernell et al.	Sweden	Matched cohort	National register	273	1 017 265	MFS	sPTB	7
Pearce et al.	UK	Cross‐sectional	Specialist clinics	947	591 072	Hypermobile EDS	PTB	5
Van den Bersselaar et al.	Netherlands	Retrospective cohort	Specialist centers	43		Vascular EDS	PTB/PPROM	6
Wright et al.	USA	Retrospective cohort	Administrative database	7378	18 207 164	EDS unspecified	PTB	9
Castori et al.	Italy	Retrospective cohort	Specialist clinic	82		Hypermobile EDS	PTB/PPROM	6
Hugon‐Rodin et al.	France	Cohort	Tertiary center	386	*	Hypermobile EDS	PTB/PPH	6
Ramage et al.	USA	Cross‐sectional	Administrative database	135	2 682 326	MFS	PTB	9
Lind & Wallenburg	Netherlands	Retrospective cohort	Hospital cohort	46	43	EDS	PTB/PPROM	7
Sundelin et al.	Sweden	Nationwide cohort	National registers	314	1 247 864	EDS	PTB	9
Hassan et al.	Canada	Retrospective cohort	Administrative database	339	7 094 061	MFS	PTB	9
Spiegel et al.	Canada	Population retrospective	Administrative database	1042	14 512 545	EDS	PTB	9
Murray et al.	USA	Retrospective cohort	Registry	565		Vascular EDS	PTB	8
Russo et al.	USA	Retrospective cohort	Aortopathy clinic	128		MFS/LDS	PTB	6

Abbreviations: EDS, Ehlers–Danlos syndrome; LDS, Loeys–Dietz syndrome; MFS, Marfan syndrome; PPH, postpartum hemorrhage; PPROM, preterm prelabour rupture of membranes; PTB, preterm birth; sPTB, spontaneous preterm birth.

* Published data from the general French population was used as a comparison, but not a control group.

Study design, population size and outcome reporting varied considerably across the included studies. Eight studies were retrospective cohort studies, three were prospective cohorts, two were cross‐sectional studies and one was a case–control study. Sample sizes ranged from 24 to 7378 pregnancies or births among women with inherited collagenopathies. Outcomes were reported inconsistently, with some studies presenting results per pregnancy, others per birth, and others per individual woman. Gestational age thresholds and definitions of sPTB also varied, and only six of the ten studies involving EDS reported outcomes according to disease subtype (hypermobile, classical, or vascular EDS). Detailed study characteristics and individual study findings are presented in Tables S5 and S6.

### 3.3 Frequency of sPTB

The frequency of sPTB was analyzed using data from studies reporting pregnancies affected by MFS or EDS. Among EDS studies, the reported frequency of sPTB ranged from 3.2% to 26.0%, whereas MFS studies reported frequencies ranging from 10.6% to 48.0% (Table S6).

Among women with vascular EDS, reported sPTB rates ranged from 14.0% to 19.1%. In hypermobile EDS, reported rates ranged from 6.2% to 15.5%. However, interpretation of these findings is limited by substantial clinical heterogeneity. Four studies did not distinguish between EDS subtypes, instead reporting outcomes for women with EDS as a single diagnostic group, while diagnostic criteria, outcome definitions and gestational age thresholds varied considerably across studies. Consequently, quantitative synthesis of EDS studies was considered exploratory only (Figures S1 and S2), and the evidence was synthesized primarily narratively.

### 3.4 Secondary maternal and neonatal outcomes

Secondary maternal and neonatal outcomes were reported inconsistently across the included studies, precluding quantitative synthesis. Five studies reported higher rates of PPROM among women with inherited collagenopathies, particularly those with EDS.^15^, ^16^, ^17^, ^18^, ^19^ Five studies also reported an increased frequency of PPH,^14^, ^16^, ^18^, ^19^, ^20^ while five observed higher rates of caesarean section,^14^, ^20^, ^21^, ^22^, ^23^ reflecting both obstetric complications and planned delivery in women with high‐risk connective tissue disorders.

Studies of vascular EDS also reported higher rates of severe obstetric complications, including cervical insufficiency, pregnancy‐related tissue fragility, and maternal death.^14^, ^20^, ^24^ In women with hypermobile EDS, reported complications more commonly included severe perineal trauma, poor wound healing and pelvic organ prolapse.^14^, ^18^, ^20^

Additional reported adverse outcomes included low birthweight,^21^ SGA infants,^21^, ^22^, ^25^, ^26^ IUGR,^22^ prolonged maternal hospitalization,^22^ and maternal death.^14^, ^20^, ^22^, ^24^ However, these outcomes were reported inconsistently, with considerable variation in definitions, outcome measures and collagenopathy subtypes, limiting meaningful comparisons across studies (Tables S5 and S6).

### 3.5 Meta‐analysis

Quantitative synthesis was restricted to studies of MFS as these represented the only clinically and methodologically comparable subgroup with sufficient data to support meaningful pooled analysis. The pooled fixed‐effect meta‐analysis demonstrated that women with MFS had significantly higher odds of sPTB than controls (OR 2.71, 95% CI 2.23–3.30; *p* < 0.001), with moderate statistical heterogeneity (*I* ^2^ = 41%) (Figure 2).

**FIGURE 2:**
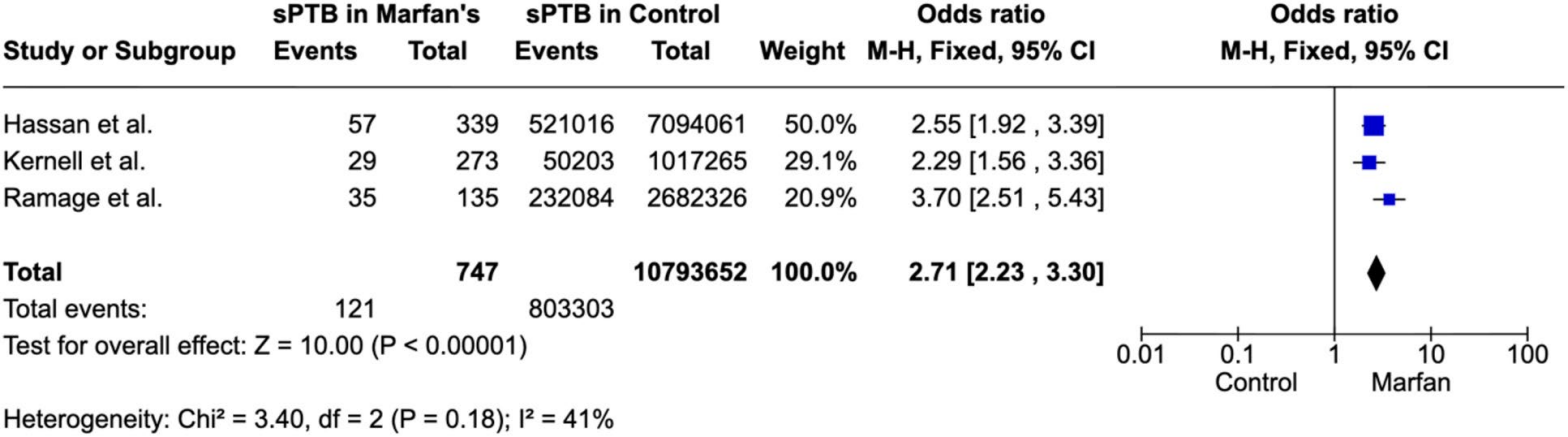
Forest plot of sensitivity for sPTB in women with MFS versus the general population (control). Odds ratios with 95% confidence intervals (CI) are shown. MFS, Marfan syndrome; sPTB, spontaneous preterm birth.

Studies of EDS were synthesized narratively because of substantial heterogeneity in subtype classification, diagnostic criteria and outcome reporting. Exploratory random‐effects analyses of EDS cohorts and pooled inherited collagenopathies are presented in the Figure S1 for transparency and completeness. However, both analyses demonstrated considerable clinical and statistical heterogeneity (*I* ^2^ = 96%–97%) and did not provide robust pooled estimates. They should therefore be interpreted with caution, are considered hypothesis‐generating only, and were not used to inform the primary conclusions of this review.

### 3.6 Quality assessment

Methodological quality varied across the included studies, with NOS scores ranging from 4 to 9 (Table S1). Five studies achieved the maximum score of 9, while most scored between 6 and 8, indicating generally adequate selection and outcome assessment but variable comparability and exposure ascertainment. Quality assessment informed interpretation of the findings and decisions regarding quantitative synthesis but was not used as the sole criterion for study inclusion.

## 4 DISCUSSION

This systematic review found that women with MFS have an increased risk of sPTB. For EDS, the available evidence remains heterogeneous, with differences in subtype classification, diagnostic criteria, and outcome reporting precluding robust quantitative synthesis. Although several studies reported higher rates of sPTB and related obstetric complications, the available evidence is insufficient to draw firm conclusions regarding the magnitude of this association.

Although the evidence remains heterogeneous, particularly for EDS, the reported associations are biologically plausible. Abnormalities in collagen and extracellular matrix proteins may impair cervical and fetal membrane integrity, providing a potential mechanism for sPTB. However, further prospective studies are needed to better define this relationship across individual collagenopathy subtypes.

Within vascular EDS, limited evidence also suggests that fetal genotype may contribute to pregnancy risk. Two studies reported that approximately half of preterm offspring carried the familial pathogenic *COL3A1* variant, although these findings are based on small cohorts and require confirmation in larger studies.^17^, ^27^ Similarly, Underhill et al.^15^ reported that infants with EDS were more likely to be born preterm than their unaffected siblings, further supporting the complex and multifactorial nature of these disorders.

Beyond sPTB, studies of MFS also reported higher rates of caesarean birth and low birthweight or SGA infants.^21^, ^25^ Women with MFS were also more likely to be primiparous. Evidence regarding maternal complications was less consistent: Hassan et al.^22^ reported maternal deaths, whereas other studies found no increase in maternal morbidity.^25^ In EDS, the pattern of adverse pregnancy outcomes also varied across studies, likely reflecting differences in subtype, phenotype, and sample size.

Previous literature has described an association between inherited collagenopathies and sPTB, largely through proposed mechanisms of cervical insufficiency and membrane fragility. However, uncertainty has remained regarding the magnitude and consistency of this association across different collagenopathy subtypes. Rather than supporting a single pooled estimate across clinically distinct disorders, our review suggests that the available evidence should be interpreted according to phenotype. Studies of MFS were sufficiently comparable to support quantitative synthesis, whereas the marked clinical and methodological heterogeneity of EDS currently limits meaningful quantitative pooling. These findings are consistent with the 2025 European Society of Cardiology Guidelines, which recommend multidisciplinary counseling and individualized risk assessment for women with vascular EDS and MFS, reflecting a move towards more personalized, evidence‐informed care.^28^ Strengths of this review include comprehensive literature searches across multiple databases (Table S2), strict inclusion criteria and structured methodological quality assessment using the Newcastle–Ottawa scale (Table S3).

The principal limitation of this review is the substantial clinical and methodological heterogeneity of the available literature, particularly among studies of EDS. Differences in subtype classification, evolving diagnostic criteria, study design and obstetric outcome definitions limited meaningful quantitative synthesis, requiring a predominantly narrative approach for EDS. This should not be interpreted as evidence that no association exists, but rather as a reflection of the current limitations of the available evidence.

Additional limitations include the small size of many study cohorts, reflecting the rarity of inherited collagenopathies, incomplete reporting of outcomes and extractable data, reliance on national administrative databases^13^, ^16^ and overlapping datasets within HCUP, which required the exclusion of one study to avoid double counting of participants.^13^ Variation in disease severity, phenotype and genetic contributions beyond *COL3A1* mutations was rarely explored, and clinically important distinctions such as spontaneous and medically indicated preterm birth were not reported consistently. Publication bias also remains a possibility, particularly if studies reporting null findings were less likely to be published.

Although most studies were rated as good quality using Newcastle–Ottawa scale, several were limited by retrospective observational designs, inconsistent outcome reporting and incomplete adjustment for important confounders, increasing the risk of residual confounding (Table S1). The limited number of studies contributing to the quantitative synthesis also precluded meaningful subgroup analyses according to study design. Collectively, these limitations should be considered when interpreting the findings and highlight the need for larger prospective studies using standardized diagnostic criteria, phenotype classification and outcome definitions across individual collagenopathy subtypes.

Overall, the available evidence suggests an increased risk of sPTB in women with MFS, whereas the evidence for EDS remains heterogeneous, with differences in subtype classification, diagnostic criteria and outcome reporting limiting firm conclusions. Although these inherited connective tissue disorders are rare and many affected women are already managed within specialist obstetric services, recognizing their association with sPTB remains clinically important. Our findings do not support the incorporation of collagenopathies into universal prediction models for sPTB. Rather, they highlight the importance of phenotype‐specific risk assessment and targeted antenatal surveillance for women with known collagenopathies.

Future collaborative registries should prioritize phenotype‐specific reporting, standardized definitions of sPTB and prospective collection of maternal and fetal genetic data. This would allow more reliable quantitative synthesis and improve our understanding of pregnancy risk across individual collagenopathy subtypes.

## 5 CONCLUSION

The available evidence suggests that pregnancy risk differs across inherited collagenopathy phenotypes. The strongest comparative evidence currently exists for MFS, whereas the evidence for EDS remains limited by substantial clinical and methodological heterogeneity. These findings support increased clinical awareness and phenotype‐specific obstetric assessment while highlighting the need for larger prospective studies using standardized diagnostic criteria and outcome reporting.

## AUTHOR CONTRIBUTIONS

**Brenda F. Narice:** writing – review and editing, writing – original draft, supervision, methodology, formal analysis, data curation, conceptualization. **Kensie Treacy:** writing – review and editing, writing – original draft, methodology, formal analysis, data curation. **Denya Williams‐Goss:** screening, data curation. **Dilly O. C. Anumba:** writing – review and editing, resources, methodology.

## Supporting information


**Table S1.** Quality Assessment Using the Newcastle Ottawa Scale (case reports and case series not included).
**Table S2.** Search Strategy.
**Table S3.** Quality assessment tool criteria used.
**Table S4.** Study characteristics and reasoning of the excluded studies at the full‐text stage.
**Table S5.** Study characteristics and methodology of the 14 included studies.
**Table S6.** Study outcomes and contribution to synthesis of the 14 included studies.
**Figure S1.** Forest Plot of sensitivity for sPTB in women with Ehlers‐Danlos Syndrome versus the general population (control). Odds ratio with 95% confidence intervals (CI) are shown.
**Figure S2.** Forest Plot of sensitivity for sPTB in women with all collagenopathies versus the general population (control). Odds ratio with 95% confidence intervals (CI) are shown.

## Data Availability

The data that support the findings of this study are available from the corresponding author upon reasonable request.
