# Supplementary material for: The association between collagenopathies and the risk of spontaneous preterm birth: A systematic review with selective meta‐analysis
Source: Acta Obstet Gynecol Scand. 2026 Oct 9:10.1111/aogs.70361. Online ahead of print. doi: 10.1111/aogs.70361 (PMC13650666; doi:10.1111/aogs.70361)
Supplement: Supplementary file 1 [file AOGS-9999-0-s001.docx]

Supplemental Information

**Table S1.** Quality Assessment Using the Newcastle Ottawa Scale (case reports and case series not included)

|  | **Selection** | | | | **Comparability** | | **Outcome** | | |  |
| --- | --- | --- | --- | --- | --- | --- | --- | --- | --- | --- |
| Reference | Representative of the exposed cohort | Selection of external exposure | Ascertainment of exposure | Outcome of interest not present at start of study | Main factor | Additional Factor | Assessment of outcomes | Sufficient follow up time | Adequacy of follow up time | Total (/9) |
| Underhill et al.^15^ | * | * |  | * |  |  |  | * |  | 4 |
| Kernell et al.^16^ | * | * | * | * |  |  | * | * | * | 7 |
| Pearce et al.^17^ | * | * |  | * |  | * |  | * |  | 5 |
| Van den Bersselaar et al.^18^ | * |  | * | * |  |  | * | * | * | 6 |
| Wright et al.^19^ | * | * | * | * | * | * | * | * | * | 9 |
| Castori et al.^20^ | * |  | * | * |  |  | * | * | * | 6 |
| Hugon-Rodin et al.^21^ | * |  | * | * |  |  | * | * | * | 6 |
| Ramage et al.^22^ | * | * | * | * | * | * | * | * | * | 9 |
| Lind & Wallenburg^24^ | * | * | * | * |  |  | * | * | * | 7 |
| Sundelin et al.^25^ | * | * | * | * | * | * | * | * | * | 9 |
| Hassan et al.^26^ | * | * | * | * | * | * | * | * | * | 9 |
| Spiegel et al.^27^ | * | * | * | * | * | * | * | * | * | 9 |
| Murray et al.^28^ | * | * | * | * | * |  | * | * | * | 8 |
| Russo et al.^29^ | * |  | * | * |  |  | * | * | * | 6 |

| **Database** | **Search Terms** | **Limitations** |
| --- | --- | --- |
| PubMed | ((premature OR "premature birth" OR prematurity OR "spontaneous preterm birth") AND (collagenopath* OR "Marfan syndrome" OR "Ehlers-Danlos syndrome" OR "connective tissue disorder*") AND pregnancy) | - Inception-April 30, 2025 - Humans - English |
| Web of Science | TS=((premature OR "premature birth" OR prematurity OR "spontaneous preterm birth") AND (collagenopath* OR "Marfan syndrome" OR "Ehlers-Danlos syndrome" OR "connective tissue disorder*") AND pregnancy) | - Inception-April 30, 2025 - English |
| Scopus | TITLE-ABS-KEY((premature OR "premature birth" OR prematurity OR "spontaneous preterm birth") AND (collagenopath* OR "Marfan syndrome" OR "Ehlers-Danlos syndrome" OR "connective tissue disorder*") AND pregnancy) | - Inception-April 30, 2025 - English |
| Embase  (Ovid) | ((premature OR "premature birth" OR prematurity OR "spontaneous preterm birth").ti,ab.  AND  (collagenopath* OR "Marfan syndrome" OR "Ehlers-Danlos syndrome" OR "connective tissue disorder*").ti,ab.  AND  pregnancy.ti,ab.) | - Inception-April 30, 2025 - Humans - English |
| Cochrane Library | ((premature OR "premature birth" OR prematurity OR "spontaneous preterm birth") AND (collagenopath* OR "Marfan syndrome" OR "Ehlers-Danlos syndrome" OR "connective tissue disorder*") AND pregnancy) | - Inception-April 30, 2025 - English |

**Table S2.** Search Strategy

**Table S3.** Quality assessment tool criteria used

| Category | Criteria | Award * if |
| --- | --- | --- |
| **Selection**  (max. 4*) | Representativeness of the exposed cohort | *The study was truly representative of the average pregnant woman with a collagenopathy  *The study was somewhat representative of the average pregnant woman with a collagenopathy |
|  | Selection of the non-exposed cohort | *Controls (the general pregnant population was drawn from the same community as women with collagenopathy) |
|  | Ascertainment of exposure | *Secure record (eg surgical records, national registry, etc.)  *Structured interview |
|  | Outcome not present at start of study | *Yes |
| **Comparability**  (max. 2*) | Main factor | *Study controls for maternal age, parity, SES, etc. |
|  | Additional factor | *Study controls for additional confounders such as smoking, ethnicity, antenatal comorbidities, etc. (multivariable regression) |
| **Outcome**  (max. 3*) | Assessment of outcome | *Independent blind assessment of PTB <37 weeks  *Record linkage |
|  | Follow-up long enough | *Yes |
|  | Adequacy of follow-up | *Complete follow-up – all subjects accounted for |

**Table S4.** Study characteristics and reasoning of the excluded studies at the full-text stage

| Study | Affected (n) | Comparator (n) | Subtype | Reason for exclusion |
| --- | --- | --- | --- | --- |
| Alrifai et al.^23^ | 1016 | 5886034 | EDS | Used the same registry data (HCUP) as Wright et al^19^, causing overlap in the population. Both papers^19,23^ also reported on the same outcome (OR for caesarean delivery) over overlapping years. Wright et al^19^ was preferred due to its newer publication, larger sample size for both affected and control groups, the inclusion of postpartum follow-up, and focusing on delivery outcomes. |
| Hammond et al.^30^ | 1 | N/A | EDS | These case reports and case studies were not to be included due to low sample size, lack of generalizability, and high risk of bias. |
| Karthikeyan and Venkat-Raman^31^ | 8 | N/A | EDS |  |
| Slaoui et al.^32^ | 1 | N/A | EDS |  |
| Vlahos et al.^33^ | 1 | N/A | MFS |  |

**Table S5.** Study characteristics and methodology of the 14 included studies

| **Study** | **Country** | **Study period** | **Design** | **Recruitment setting** | **Population / Eligibility** | **Comparator** | **Collagenopathy subtype** | **Diagnostic criteria** | **Outcome definition** | **Gestational age definition** |
| --- | --- | --- | --- | --- | --- | --- | --- | --- | --- | --- |
| Underhill et al.^15^ | USA | 2016–2019 | Observational cohort, cross-sectional | Patient registry | Pregnant women with EDS | 206 controls | EDS (all types) | Not reported | sPTB/PTB | ~33 weeks |
| Kernell et al.^16^ | Sweden | 1997–2013 | Population-based matched cohort | National birth register | Women with MFS | 1,017,265 controls | MFS | Clinical diagnosis | sPTB |  |
| Pearce et al.^17^ | UK/international | 2020–2022 | Cross-sectional mixed methods | Specialist clinics | Hypermobile EDS | Population comparator | Hypermobile EDS | Clinical diagnosis | PTB |  |
| Van den Bersselaar et al.^18^ | Netherlands | 2000–2022 | Retrospective multicentre cohort | Specialist centres | vEDS pregnancies | vEDS cohort | Vascular EDS | COL3A1 confirmed | PTB | 26–34 weeks |
| Wright et al.^19^ | USA | 2016–2019 | Retrospective cohort | HCUP administrative database | Women with EDS | 18,207,164 controls | EDS (unspecified) | ICD coding | PTB |  |
| Castori et al.^20^ | Italy |  | Retrospective cohort | Specialist clinic | Hypermobile EDS | Hypermobile EDS | Hypermobile EDS | Clinical diagnosis | PTB | 26 weeks |
| Hugon-Rodin et al.^21^ | France | 2000–2015 | Cohort | Tertiary centre | Hypermobile EDS | Hypermobile EDS | Hypermobile EDS | Clinical diagnosis | PTB |  |
| Ramage et al.^22^ | Canada | 2000–2014 | Cross-sectional | Administrative database | MFS | 2,682,326 controls | MFS | ICD coding | PTB |  |
| Lind & Wallenburg^24^ | Netherlands | 1988–2000 | Population-based retrospective cohort | Hospital | EDS | 43 controls | EDS (all types) | NR | PTB |  |
| Sundelin et al.^25^ | Sweden | 1997–2011 | Nationwide cohort | National registers | EDS | 1,247,864 controls | Unclassified EDS | ICD coding | PTB |  |
| Hassan et al.^26^ | Canada | 1989–2012 | Retrospective cohort | Administrative database | MFS | 7,094,061 controls | MFS | ICD coding | PTB |  |
| Spiegel et al.^27^ | Canada | 1989–2019 | Population-based retrospective cohort | Administrative database | EDS | 14,512,545 controls | EDS | ICD coding | PTB |  |
| Murray et al.^28^ | USA | 1974–2011 | Retrospective mixed methods | Registry | vEDS | vEDS cohort | Vascular EDS | COL3A1 | PTB |  |
| Russo et al.^29^ | USA |  | Retrospective observational cohort | Inherited aortopathy clinic | MFS & Loeys–Dietz syndrome | MFS/Loeys–Dietz | MFS + Loeys–Dietz | Clinical/genetic | PTB |  |

*EDS: Ehlers-Danlos syndrome, ICD: International Classification of Diseases, MFS: Marfan syndrome, PTB: preterm birth, PPH: postpartum haemorrhage, sPTB: spontaneous preterm birth, vEDS: vascular Ehlers-Danlos syndrome*

**Table S6.** Study outcomes and contribution to synthesis of the 14 included studies

| **Study** | **Maternal outcomes** | **Neonatal outcomes** | **Adjusted analyses (covariates included)** | **Main findings** | **Contribution to synthesis** |
| --- | --- | --- | --- | --- | --- |
| Underhill et al.^15^ | PPROM, pregnancy complications | Prematurity | No | 26% PTB; affected infants more likely premature | Narrative synthesis |
| Kernell et al.^16^ | Higher CS, primiparity | SGA/LBW | Yes (maternal age, maternal/paternal education, parity, marital status, parental country of origin, smoking, BMI) | Increased sPTB (10.6%) | Primary Marfan meta-analysis |
| Pearce et al.^17^ | Rapid labour, poor wound healing | Stillbirth reported | No | PTB 15.5% | Narrative synthesis |
| Van den Bersselaar et al.^18^ | PPROM, PPH, cervical insufficiency |  | No | PTB 19.1% | Narrative synthesis |
| Wright et al.^19^ | Cervical insufficiency, PPH, CS, severe maternal morbidity | Prematurity | Yes (maternal age, hospital characteristics, insurance payer, income quartile, mode of delivery, comorbidities) | OR PTB 1.35 (1.16–1.56) | Narrative synthesis |
| Castori et al.^20^ | PPROM, prolapse, PPH |  | No | PTB 10.7% | Narrative synthesis |
| Hugon-Rodin et al.^21^ | PPH | Stillbirth reported | No | PTB 6.2% | Narrative synthesis |
| Ramage et al.^22^ | Primiparity | SGA | Yes (maternal age, parity, socioeconomic status, rural residence, hypertension, diabetes) | PTB 25.9% | Primary Marfan meta-analysis |
| Lind & Wallenburg^24^ | PPROM, PPH | Floppy infant syndrome | No | PTB 22% | Narrative synthesis |
| Sundelin et al.^25^ | Increased induction | No excess neonatal morbidity | Yes (maternal age, BMI, smoking, parity, education, country of birth, year of delivery) | PTB 3.2% | Narrative synthesis |
| Hassan et al.^26^ | Cardiac morbidity, CS | IUGR/SGA | Yes (maternal age, parity, multiple gestation, diabetes, hypertension, previous CS, obesity, smoking, comorbidities) | PTB 16.8% | Primary Marfan meta-analysis |
| Spiegel et al.^27^ | APH, CS, prolonged stay | IUGR | Yes (maternal age, parity, multiple pregnancy, hypertension, diabetes, obesity, previous CS, smoking, comorbidities) | PTB 8.9% | Narrative synthesis |
| Murray et al.^28^ | Severe tears |  | No | PTB 14% | Narrative synthesis |
| Russo et al.^29^ | High CS |  | No | PTB 48% | Narrative synthesis |

*APH: antepartum haemorrhage, BMI: body mass index, CS: caesarean section, EDS: Ehlers-Danlos syndrome, IUGR: intrauterine growth restriction, LBW: low birth weight MFS: Marfan syndrome, PTB: preterm birth, PROM: prelabour rupture of membranes, PPH: postpartum haemorrhage, SGA: small for gestational age, sPTB: spontaneous preterm birth, vEDS: vascular Ehlers-Danlos syndrome*

**Figure S1.** Forest Plot of sensitivity for sPTB in women with Ehlers-Danlos Syndrome versus the general population (control). Odds ratio with 95% confidence intervals (CI) are shown.

*EDS: Ehlers-Danlos syndrome , sPTB: spontaneous preterm birth*

**Figure S2.** Forest Plot of sensitivity for sPTB in women with all collagenopathies versus the general population (control). Odds ratio with 95% confidence intervals (CI) are shown.

References

30. Hammond R, Oligbo N. Ehlers Danlos syndrome type IV and pregnancy. *Arch Gynecol Obstet.* 2011;285(1):51-54. doi:10.1007/s00404-011-1899-5

31. Karthikeyan A, Venkat-Raman N. Hypermobile Ehlers-Danlos syndrome and pregnancy. *Obstet Med.* 2018;11(3):104-109. doi:10.1177/1753495X18754577

32. Slaoui A, Mahtate M, Lazhar H, Lakhdar A, Baydada A, Kharbach A. Spontaneous uterine rupture revealing vascular Ehlers-Danlos syndrome. *Int J Surg Case Rep.* 2022;92(c):106840. doi:10.1016/j.ijscr.2022.106840

33. Vlahos NF, Triantafyllidou O, Vitoratos N, Grigoriadis C, Creatsas G. Preimplantation genetic diagnosis in Marfan syndrome. *Case Rep Obstet Gynecol.* 2013;2013(1):1-3. doi:10.1155/2013/542961
